# Large Mesenteric Cyst Mimicking Tuberculous Ascites

**DOI:** 10.1155/2010/725050

**Published:** 2010-06-08

**Authors:** Cumhur Dulger, Ertan Adali, Serhat Avcu, Zehra Kurdoglu

**Affiliations:** ^1^Department of Gastroenterology, Yuzuncu Yil University, PK 65300, Van, Turkey; ^2^Department of Obstetrics and Gynaecology, Yuzuncu Yil University, PK 65300, Van, Turkey; ^3^Department of Radiology, Yuzuncu Yil University, PK 65300, Van, Turkey

## Abstract

*Background*. Intraabdominal lesions such as mesenteric cysts are uncommon disorders. Most are discovered incidentally during routine abdominal examinations. *Methods*. We report a patient with a mesenteric cyst masquerading as tuberculous peritonitis and ascites. *Conclusion*. Mesenteric cysts generally do not show typical clinical findings. They may also present with peritoneal tuberculosis findings such as low albumin gradient ascites with high ascitic adenosine deaminase levels. Surgery is used to remove a wide variety of mesenteric cysts. A correct diagnosis can be made by the combined use of radiographic and sonographic examinations in conjunction with the clinical signs.

## 1. Introduction

Mesenteric cysts (MCs) are rare intra-abdominal lesions arising with an incidence of 1/100,000 admissions in adults and 1/20,000in children [1]. Many authors consider mesenteric, omental, and retroperitoneal cysts as one group because they derive from the same embryological structures [2], whereas others have defined MCs as cysts arising in or near the mesentery with no connection to retroperitoneal organs [1]. The symptoms are variable and nonspecific and include pain (82%), nausea and vomiting (45%), constipation (27%), and diarrhea (6%). An abdominal mass may be palpable in up to 61% of patients [3]. Herein we present a patient with a huge MC mimicking ascites and peritoneal tuberculosis.

## 2. Case Report

**Figure 1:**
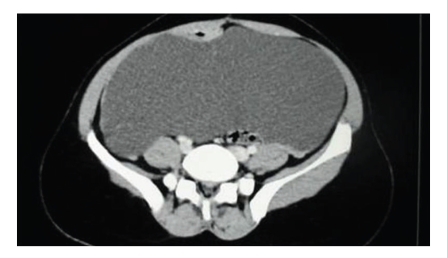
Appearance of large mesenteric cyst on abdominal CT.

**Figure 2:**
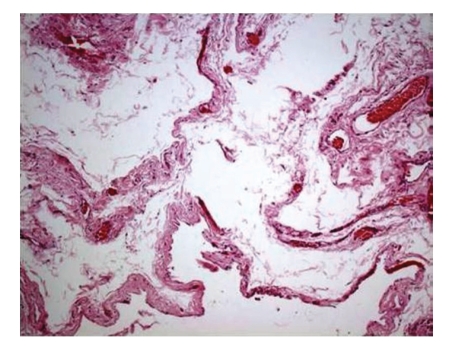
Mesenteric cyst. Cystic structures lined by endothelium-like attenuated cells and thin cystic walls are seen (H&E stain, original magnification: x10 objective).

A 25-year-old woman was admitted to our hospital with an 8-month history of abdominal pain, distension, intermittent nausea, vomiting, and low-grade fever. She had undergone abdominal surgery due to an accidental gunshot injury 10 years earlier. She had ascites on physical examination. Laboratory examinations were unremarkable and the upright abdominal X-ray was normal. Abdominal ultrasound (US) showed multiloculated ascites. A computerized tomographic (CT) scan showed ascites with thin intraascitic septations. The serum-ascites albumin gradient was 0.4 g/dL and the adenosine deaminase level of ascites was 49 U/l (upper limit of normal: 40 U/l). Moreover, the ascitic fluid was straw colored with protein >3 g/dL and total cell count of 550 /*μ*L, consisting predominantly of lymphocytes (>70%). Tumor markers were with the normal range in serum but the ascitic CA-125 level was 336 U/mL (upper limit of normal: 16 U/mL). Cultures of the ascitic fluid and blood were negative for mycobacterial colonies. She refused to undergo laparoscopy, most likely due to her busy schedule. The findings were compatible with peritoneal tuberculosis and conventional antitubercular therapy was started. She returned to the hospital six months later. At the time of her second admission, she was well but her abdominal dullness and ascites were persisting. She had not taken any kind of medication for four months. Levels of serum electrolytes, lactate dehydrogenase, creatine kinase, albumin, and total protein and results of renal- and liver-function tests were normal. Repeated abdominal and pelvic CT scanning with contrast medium showed a large MC (Figure 1). Exploratory laparotomy revealed a multiloculated thin-walled cyst arising from the colonic mesentery, adherent to the bowel loops and adjacent structures. During dissection of adhesions some of the locules ruptured, and about 5 liters of hemorrhagic fluid were suctioned. Histological examination of the biopsy specimen revealed an MC (Figure 2). The postoperative course was very good and the patient was discharged.

## 3. Discussion

Although MCs can occur at any age, they are common in people between 40 and 70 years old; however, they also affect children younger than 10 years old. MCs usually range in size from a few centimeters to 10 cm but they can also be very large [4]. The most frequent site is the mesentery (60%), followed by the mesocolon (24%) and the retroperitoneum (14.5%), while it is indefinite in 1.5% of cases [5]. In our case ultrasonography showed multiloculated fluid collection with internal echoes in the abdomen. 

Small MCs may be asymptomatic but large cysts can present as acute abdominal emergencies such as acute intestinal obstruction, or volvulus, or sudden hemorrhage into the cyst, or infection [6]. In our case, abdominal discomfort and dullness were the main symptoms that prompted the patient to seek treatment. The cysts can remain asymptomatic and therefore grow to giant proportions, as illustrated in the present case. 

The cyst often has a flaccid consistency and tends to “flow out” and “fill out” the dependent parts of the abdominal cavity and interposes between the structures. Usually the diagnosis of mesenteric cyst is made after the exclusion of other diseases [6]. 

With the development of US and CT, the preoperative diagnosis of abdominal cystic disorder has become the rule. US can distinguish between solid and cystic masses and CT can determine extension and cystic content [7, 8]. On US, mesenteric cysts tend to appear as cystic structures, usually with internal septations as in our case. The CT scan of the abdomen failed to demonstrate the cyst due to its large size at her first admission. 

MCs are rare and benign lesion. Although physical examination and abdominal US indicate remarkable ascites, giant MCs may be mistaken for ascites on physical examination and imaging studies [9]. Therefore, this diagnosis should be included in the differential diagnosis of abdominal distension, even when radiological examination (US or CT) indicates ascites. In our case, the mesenteric cyst was giant, and it was mimicking ascites on the initial computed tomography which was obtained without administration of contrast medium. But upon following computed tomography examination with intravenous contrast medium, the thin wall of the mesenteric cyst is better demonstrated, and a correct radiological diagnosis of the lesion could be maintained.

Adenosine deaminase (ADA) is an aminohydrolase that converts adenosine to inosine; so it is a purine-degrading enzyme and allows for preliminary diagnosis of peritoneal tuberculosis. Previous studies have demonstrated that ADA (cutoff 30–33 U/l) has a sensitivity, specificity, and diagnostic accuracy in peritoneal tuberculosis that were 100%, 97%, and 98%, respectively [10, 11]. A Turkish study has described that 83% of TP patients had elevated levels of ADA (mean : 65 U/l) [12]. ADA activity determination in ascitic fluid is a useful noninvasive screening test in the diagnosis of peritoneal tuberculosis in endemic areas and endorses empirical initiation of antitubercular therapy pending confirmatory tests. So, we considered abdominal tuberculosis because of high ascitic ADA levels and because of higher endemicity in our region. So, we started antituberculosis therapy based on these reasons. Furthermore, she refused to undergo laparoscopy at initial admission. However, the lack of response to therapy, absence of mycobacterial culture findings, and finally the lack of caseating granulomas did not support this diagnosis. The low serum-ascites albumin gradient with slightly elevated ADA levels may not be sufficient for the diagnosis of tuberculosis peritonitis. There are several case reports of elevated CA-125 levels in women with M. tuberculosis infection of the peritoneal cavity [13]. In these patients, the initial CA-125 levels were often higher than 500 U per milliliter. In our case, the ascites level of CA-125 was 336 U/mL and was consistent with peritoneal tuberculosis. This case highlights the difficulty in establishing a diagnosis of peritoneal mycobacterial infection. This difficulty is further compounded by intraperitoneal strictures of patient and higher endemicity of disease in our provinence.

The treatment of choice is complete excision to avoid recurrence and possible malignant transformation [14]. However, this was not feasible in our patient because of extensive adhesions to the bowel due to a previous accidental gunshot injury. The postoperative period was clinically uneventful with no signs or symptoms of complications. With the exception of malignant cystic mesotheliomas, all MCs are benign and their total excision is usually curative. However, benign cystic mesotheliomas and lymphangiomas have a high tendency to recur if they are incompletely resected [15]. Therefore we recommended closer observation for her. 

In conclusion, MCs are rare benign abdominal tumors. The pathogenesis is not uniform and the clinical and radiologic diagnosis is difficult. The symptoms of this condition vary from acute abdominal signs to nonspecific abdominal features or incidental findings. With an accurate history, a thorough clinical examination, a high index of suspicion, and the use of radiological examination, a definitive diagnosis can be made. The thin wall of the MCs can be better demonstrated with CT examination after intravenous contrast medium, and a correct radiological diagnosis of the lesion can be maintained.
